# Application of Machine Learning Techniques to an Agent-Based Model of *Pantoea*

**DOI:** 10.3389/fmicb.2021.726409

**Published:** 2021-09-24

**Authors:** Serena H. Chen, Pablo Londoño-Larrea, Andrew Stephen McGough, Amber N. Bible, Chathika Gunaratne, Pablo A. Araujo-Granda, Jennifer L. Morrell-Falvey, Debsindhu Bhowmik, Miguel Fuentes-Cabrera

**Affiliations:** ^1^ Computational Sciences and Engineering Division, Oak Ridge National Laboratory, Oak Ridge, TN, United States; ^2^ Chemical Engineering Faculty, Universidad Central del Ecuador, Quito, Ecuador; ^3^ School of Computing, Newcastle University, Newcastle upon Tyne, United Kingdom; ^4^ Biosciences Division, Oak Ridge National Laboratory, Oak Ridge, TN, United States; ^5^ Computer Science and Mathematics Division, Oak Ridge National Laboratory, Oak Ridge, TN, United States; ^6^ Center for Nanophase Materials Sciences, Oak Ridge National Laboratory, Oak Ridge, TN, United States

**Keywords:** agent-based model, machine learning, random forest regression, neural network, *Pantoea*

## Abstract

Agent-based modeling (ABM) is a powerful simulation technique which describes a complex dynamic system based on its interacting constituent entities. While the flexibility of ABM enables broad application, the complexity of real-world models demands intensive computing resources and computational time; however, a metamodel may be constructed to gain insight at less computational expense. Here, we developed a model in *NetLogo* to describe the growth of a microbial population consisting of *Pantoea*. We applied 13 parameters that defined the model and actively changed seven of the parameters to modulate the evolution of the population curve in response to these changes. We efficiently performed more than 3,000 simulations using a Python wrapper, *NL4Py*. Upon evaluation of the correlation between the active parameters and outputs by random forest regression, we found that the parameters which define the depth of medium and glucose concentration affect the population curves significantly. Subsequently, we constructed a metamodel, a dense neural network, to predict the simulation outputs from the active parameters and found that it achieves high prediction accuracy, reaching an *R*^2^ coefficient of determination value up to 0.92. Our approach of using a combination of ABM with random forest regression and neural network reduces the number of required ABM simulations. The simplified and refined metamodels may provide insights into the complex dynamic system before their transition to more sophisticated models that run on high-performance computing systems. The ultimate goal is to build a bridge between simulation and experiment, allowing model validation by comparing the simulated data to experimental data in microbiology.

## Introduction

The Earth may be described as a microbial planet with microbes found in complex communities that perform a wide range of important functions, from microbial communities in the human gut, to communities in soil that affect the growth of plants (Leveau et al., 2018). Although, advances in genomics, metabolomics, and imaging have provided clues about the organization and composition of microbial communities, our ability to predict and manipulate the functions of microbial communities for desired outcomes is limited (Kreft et al., 2017). Complementing these experimental approaches, simulation techniques, such as agent-based modeling (ABM), are moving to the forefront as a powerful tool to unravel how interactions between microbes lead to emergent traits at the community level (Kreft et al., 1998, 2017; Hellweger et al., 2008, 2016; Leveau et al., 2018).

As with any simulation technique, the accuracy of an ABM is determined by comparing the simulation data to the experimental data, a process known as validation. In microbiology, the experimental data can exist in many forms, such as genomic sequences, metabolite profiles, growth curves of individual microbial strains, or images of microbial growth. Validating the ABM in a multi-scale manner is possible using pattern oriented modeling (POM), but it is also very expensive computationally (Grimm et al., 2005). To reduce the computational burden, it is customary to first perform a sensitivity analysis (SA) of the ABM (Ligmann-Zielinska, 2013; Ligmann-Zielinska et al., 2014; Thiele et al., 2014; ten Broeke et al., 2016). With SA, one can uncover which parameters of the model have the strongest effect on the output, and what correlations, if any, exist among them. Then, during the validation process, one can focus on varying the independent parameters only, thereby reducing the computational burden.

We have started building ABMs that are capable of reproducing experimental local and global information of microbial growth. Local information can be size and shape of the colonies, length of the boundaries between colonies, etc. Global information is usually represented by the population curves of the whole community, where growth is monitored over time. As part of this effort, we have focused on investigating the microbial interactions in the rhizosphere of poplar trees (*Populus deltoides* or *Populus trichocarpa*), a tree that shows promise as a bio-energy crop (Cregger et al., 2021). *Pantoea* sp. YR343 is a gamma-proteobacterium that was isolated from the rhizosphere of *P. deltoides* and possesses several plant growth-promoting characteristics (Bible et al., 2016). In the work presented here, we developed the ABM to investigate the growth of *Pantoea*. This model is an improvement to the previous model made for investigating a population containing a wild-type and a mutant strain of *Pseudomonas aeruginosa* (Wilmoth et al., 2018).

The ABM developed is written in the language *NetLogo* (Wilensky, 1999). This language was designed for teaching complex phenomena to students and thus it is easy to learn. This has undoubtedly contributed to its popularity. Further, we have found that *NetLogo’s* interface allows experimentalists to run simulations and explore different scenarios, facilitating the exchanges of ideas and further improvement of the model. Nonetheless, in general, *NetLogo* lacks capabilities for performing simulations on high-performance computing systems, although, recent works have shown that significant improvements in performance can sometimes be attained (Ayllón et al., 2016; Railsback et al., 2017). Despite this, *NetLogo* can be a powerful tool for prototyping ideas before implementing them in more sophisticated software, such as NUFEB (Li et al., 2019).

The model present here contains 13 parameters, thus making the SA and validation analysis computationally prohibitive. This limitation forced us to turn our attention to identifying ways to simplify our model that circumvent the need to carry out a large number of simulations (Pereda et al., 2017). To this end, we turned our attention to machine learning (ML), specifically deep learning (DL). ML and particularly DL have undergone significant advances in the last 10years. Using application programming interfaces (APIs) such as *Keras* (Chollet, 2015) and scientific packages such as *SciPy* (Virtanen et al., 2020), it is relatively simple to write code to classify objects, perform regression, or make predictions. The Web is also full of blogs and courses that flatten the learning curve for these types of techniques. ML and DL have also been applied to microbiology, yet the confluence of these techniques with ABMs is, to our knowledge, not broadly used (Lee et al., 2020).

Here, the population curves of *Pantoea* produced by the *NetLogo* ABM are compared to the experiments. The simulated population curves are also analyzed using random forest regression to study the correlation between the parameters and outputs of the ABM and pinpoint the most important parameters of the model. Then, we build a fully dense neural network, which we show is capable of producing results statistically similar to that of the original ABM. The choice of these ML techniques is due to their consistent robustness and accuracy in prediction (Biau, 2012; Abiodun et al., 2018; Couronné et al., 2018). To improve the efficiency of our ABM simulations, we use *NL4Py* (Gunaratne and Garibay, 2018), a Python wrapper for *NetLogo*, which permits submitting thousands of simulations in a parallel fashion. We also note challenges when comparing simulated and experimental population curves, and we suggest how to circumvent this limitation in the Discussion section.

## Materials and Methods

### Agent-Based Modeling Method

The ABM was designed to reproduce the behavior of a *Pantoea* growing in a *R2A*-rich agar petri dish. This model uses many of the implementations in IBM-INDISIM (Ginovart et al., 2002), the ABM which has been successfully used to simulate microbial cultures in situations such as fermentation, multi-species composting, and yeast dynamics in aerobic media and denitrification processes (Ginovart and Cañadas, 2008; Prats et al., 2010; Portell et al., 2014; Banitz et al., 2015; Araujo Granda et al., 2016b). As in IBM-INDISIM, we use a thermodynamic approach to describe microbial metabolism, e.g., cellular maintenance and mass production, in the ABM for *Pantoea*. This approach is called the *Thermodynamic Equivalent Electron model* (TEEM), and it relies on a set of thermo-chemical reactions that account for the Gibbs free energy involved in the overall metabolism, catabolism, and anabolism (McCarty, 2007). We also assume that the growth is Monod driven with a maximum growth rate, μ_max_, ranging from 0 to 10.

Two types of entity exist in our ABM: the bacterium and the square patches where it moves and grows. Each bacterium has its own unique identification number, biomass, metabolism, and reproduction parameters, as well as viability and location coordinates, and each one performs the following actions: nutrient uptake, cellular maintenance, biomass synthesis, product generation, and bipartition (reproduction). The time-dependent variables are calculated and updated in each time-step according to a time scale of 0.1–1.6min per time-step. The square patches contain the R2A agar growth medium, and growth medium actions include changes in metabolite concentration and nutrient consumption on each patch. Carbon and nitrogen consumption, as well as their generation and accumulation, are controlled to ensure mass balance. Additionally, the model includes behavior actions that control the overlap of bacteria and the interaction after bipartition.

The empirical formula C_4.17_H_8_O_1.75_N (Araujo Granda et al., 2016a) was used to describe the elemental composition of *Pantoea*. The bacterium’s dimensions were assumed to have length and width at 1.1±0.5μm and 0.55±0.25μm (mean±SD), respectively (Kapetas et al., 2012). The individual mass was deduced from the volume and an assumed density of 1.1g/cm^3^ (Gras et al., 2011). A two-dimensional lattice grid was used to describe the environment where the bacteria grow. Each grid-cell represents a volume that can be tuned by changing the *world dimensions*, depth, and length. A volume was calculated using a portion of the wells in the experimental setup with dimensions of 605μm×605μm×*depth*, where *depth* ranges from 10 to 50μm. The initial concentration of glucose and ammonium were estimated from commercial *R2A* agar composition.

When doing simulations with ABMs, it is important to produce a large number of simulations. This is because ABMs are stochastic, and many simulations are needed to obtain reliable statistics. We used *NL4Py* to investigate 3,358 different initial configurations. *NL4Py* (Gunaratne and Garibay, 2018) is a Python library that facilitates the control and reporting of parallelizable *NetLogo* workspaces. This allowed us to interface ML algorithms implemented in the *SciKit-Learn* (Pedregosa et al., 2011) and *Keras* (Chollet, 2015) Python libraries with the *NetLogo* model and provide automated simulation deployment and evaluation. For each of the 3,358 initial configurations, we ran 4–6 repetitions to ensure statistical sampling, which yields around 18,000 simulations in total. In these simulations, we fixed the values of six parameters to values generally accepted by the literature or based on our experiments: efficiency (McCarty, 2007), energy_maintenance_pa (Gras et al., 2011), ammonium (Wushensky et al., 2018), total-length-world based on the experiments described above, and lastly, max-time-viability_pa and rep_pa based on preliminary tests following a similar application by Font Marques and Ginovart (2016). The remaining seven parameters, namely pmax, microorganism, depth, umax_pa, diffusion-coefficient, glucose, and min/steptime, were randomly varied within a pre-defined range. A brief description of the 13 parameters and their values that define this ABM is listed in Table 1. The Overview-Design Concepts-Details (ODD; Grimm et al., 2010, 2020), the *NetLogo* implementation, and the Python code for the simulations using *NL4Py* are available at https://github.com/miguel-fc/ABM-Pantoea.

**Table 1:** Parameters in the agent-based modeling (ABM).

Parameters	Description	Values [increment] (units)
*Efficiency*	Thermodynamic reactions efficiency	0.37 (–)^*^
*umax_pa*	Maximum growth rate of *Pantoea* individuals	0.1–10 [0.1] (h^−1^)
*min/steptime*	Time-scalable conversion from ticks to minutes	0.1–2 [0.5] (min)
*diffusion-coefficient*	Rate of redistribution of nutrients on each patch at each steptime	0–1 [0.01] (–)^*^
*energy_maintenance_pa*	Minimum carbon needed by *Pantoea* to survive in time	0.0015 (gC_glucose_·gC_pantoea_^−1^·h^−1^)
*rep_pa*	Minimum time needed by *Pantoea* to get ready for reproduction	20 (min)
*max-time-viability_pa*	Maximum time that *Pantoea* can survive without its minimum energy requirements	83 (min)
*pmax*	Maximum number of bacteria allowed in the same patch	1–10 [1] (#·patch^−1^)
*Ammonium*	Initial concentration of ammonium in patches	18.7 (mM)
*Glucose*	Initial concentration of glucose in patches	0–100 [0.1] (mM)
*Total-length-world*	Value of world width and world height	605 (μm)
*Depth*	Depth of the first “layer” of medium where the bacteria grow	10–50 [1] (μm)
*Microorganism*	Initial number of *Pantoea* individuals	10–1,000 [1] (#)

* –, dimensionless.

**Figure 1:**
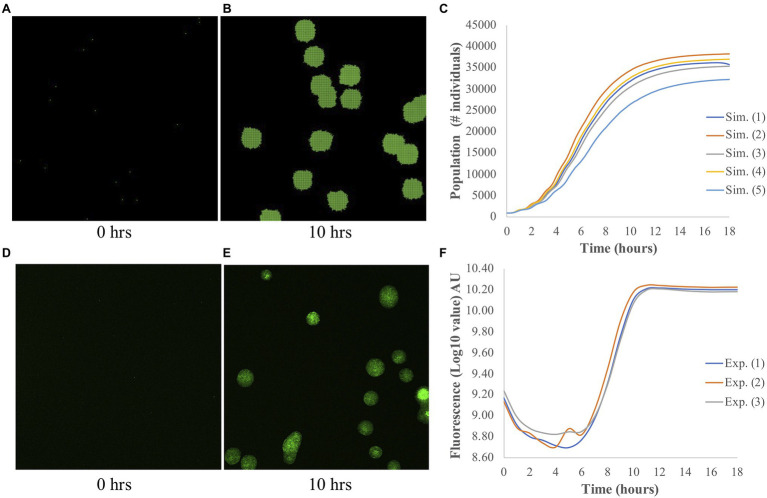
Simulated and experimental population growth of *Pantoea*. **(A–C)** Simulated growth. Snapshots of simulated microbial population at **(A)** *t*=0h and **(B)** *t*=10h. **(C)** The corresponding population curve, which shows the typical logistic shape. **(D–F)** Experimental growth. Representative images of bacterial colony growth at **(D)** *t*=0h and **(E)** *t*=10h. Images were taken from individual wells for measurements of fluorescence intensity. **(F)** The corresponding population curve shows the changes in fluorescence intensity over time. Fluorescence is measured from *Pantoea* sp. YR343 expressing GFP and is used to measure the growth of these cells over time. Note the different *y*-axes of **(C)** and **(F)**.

The output of each simulation consisted of a population curve, which shows how much the population grew over the simulation time. As described by Wilmoth et al. (2018), we fit the growth part of each population curve to a logistic function. From this fitting, three parameters, namely the maximum (A), the slope (*μ*), and the lag-time (*τ*), were obtained, and they are used to identify each population curve. An example of a simulated image of bacterial growth at *t*=0h and *t*=10h, and a population curve are shown in Figures 1A–C, respectively.

It is important to explain why we chose fitting our population curves to a logistic function. Doing this fitting seems counterintuitive, given that by assuming from the onset that *Pantoea* grows in a Monod fashion, we already know the shape of the population curves even without running the simulations. When a Monod growth is assumed from the onset, the power of running ABM simulations relies not in determining the growth of a whole population, of course, but on determining the local growth, i.e., at the level of a few bacteria. At the local level, nutrient concentration and microbe-microbe interactions are bound to affect the growth in a different way that they do at a global population level. Determining these differences, though, requires analyzing the images of growth, and extracting metrics that correlate microbe-microbe proximity and/or nutrient concentration to growth; this is out of the scope of the present work. The purpose here is to find a method for refining and simplifying our model. It is for this reason that we decided to focus on the population level only, and assumed we knew nothing about its growth. In such a scenario, it is common to fit the population curves to different functions. We used here one of these functions (Zwietering et al., 1990) and used the parameters that define the curves as the outputs of our simulations. By studying the effect of the inputs of the ABM on these outputs, we are then able to understand our model better and suggest refinements.

### Experimental Methods

Image-based growth curves were performed using a glass bottom 24-well black plate (CellVis, catalog P24-1.5H-N) with the BioTek Cytation 5 high content imaging plate reader. In order to visualize bacterial colony growth, we used R2A media with 1.5% agarose to make thin agar pads (300μl spread across the well) in each well of the plate and allowed them to solidify. We next prepared a culture of *Pantoea* sp. YR343 expressing GFP by growing them overnight in R2A media (OD at 600nm was approximately 1), then diluting the culture with fresh R2A media to 10^−4^ cells/ml. From the diluted culture, we added 300μl of cells to each well and incubated the plate at 28°C for 1h. Next, we removed any excess media and allowed the plate to dry for 1h at 28°C. After that, we placed the 24-well plate in the Cytation 5 and set imaging parameters to take images and measure fluorescence intensity every hour for a total of 18h. Fluorescence intensity values were used to generate growth curves. See Figures 1D–F for an example of experimental results.

In experimental studies, we observed a lag phase that lasted approximately 6–8h post inoculation, followed by a period of exponential growth that lasted 3–4h, and ending in stationary phase. Parameters from these studies, such as media composition, volume of the well, and growth rates were used to inform the simulation studies.

### Random Forest Regression

We used Random Forest Regression *via* the *Scikit-Learn* Python Library (Pedregosa et al., 2011) to understand the correlation between the seven input parameters and the three outputs. We first sorted the 3,358 population curves into four subsets based on their steptime values. The four steptime values used in the simulation were 0.1, 0.6, 1.1, and 1.6min, which had 831, 837, 879, and 811 population curves, respectively. Next, for each subset, we randomly split the corresponding population curves into two datasets, training and test, in a 70/30 ratio. Each population curve in a steptime subset was associated with a unique set of the remaining six input parameters and average values of the three outputs. As the inputs are on different scales, we normalized each of their distributions to zero mean and unit variance to allow for efficient training. For each output, we first constructed a Random Forest Regression model and optimized its hyperparameters by random search (Bergstra and Bengio, 2012) with 3-fold cross validation on the training dataset. The hyperparameters that were tuned included the number of decision trees in the forest, the maximum depth of the tree, the minimum number of samples required to split an internal node and to be at a leaf node, the number of features considered by each tree to split a node, and whether or not bootstrap samples when building trees. We then used each optimized model to predict the corresponding output of the test dataset and evaluated the models by their coefficients of determination, *R*^2^ scores, between the predicted and simulation (true) outputs.

### Neural Network

The methodology above leads to one Random Forest Regression model for each of the three outputs at a particular steptime value. With a feed-forward Neural Network, we aim to build one single comprehensive model that is not only capable of predicting the three outputs simultaneously but also invariant to steptime. Therefore, we considered all 3,358 population curves and randomly split them into training and test datasets in a 70/30 ratio. We built the network using the *Keras* Library (Chollet, 2015). The network was composed of two hidden layers with 30 and 15 nodes, respectively. The output layer had three nodes, one for each output. The total number of trainable parameters was 753. We used a ReLU activation function at each hidden layer, mean squared error as the loss function and the Adam optimizer with a learning rate of 0.01. As the inputs and outputs are on different scales, we normalized each to values between 0 and 1. We evaluated the network using 3-fold cross validation, each fold for 300 epochs and a batch size of 50. The mean absolute error (MAE) over the three folds was 0.018, with a SD of 0.001. We then re-trained the network on the entire training dataset and validated it on the remaining test dataset using the same set of hyperparameters found in the cross validation. The MAE was 0.019. To calculate the coefficients of determination, *R*^2^ scores, of the three outputs, we applied the final network on the test dataset and transformed the predicted values back to the original scales.

## Results

*Pantoea* sp. YR343 is a robust colonizer of plant roots in the rhizosphere, where it competes with many other microbes for both space and resources. In order to better understand how competition for resources affects the spatial distribution of bacterial growth, we used microscopy to observe growth of bacterial colonies on an agar surface in order to develop the simulation tools described in this study. Experimental growth curves showed an initial lag time of approximately 8h before entering into exponential phase, where *Pantoea* sp. YR343 forms small, mucoid colonies that grow rapidly, and at later timepoints, begin merging with other colonies before they enter stationary phase. Data obtained from the experimental growth curves were used to inform the parameters for our simulations.

To assess the accuracy of our ABM for *Pantoea*, we compared the populations curves from simulation and experiment; examples of these curves are shown in Figure 1. By fitting the growth curves to a logistic function, we extracted the three outputs: the maximum A, the slope *μ*, and the lag time *τ*. Both experiments and simulations have comparable *μ*, in which the averaged values are 5.12±0.95h^−1^ and 6.08±0.78h^−1^ (±SD), respectively. However, *τ* is shorter in simulations, with an average value of 2.62±0.13h, as compared to 8.29±0.17h in experiments. The difference in the experimental and simulated values for *τ* occurs because in the experiments, *τ* was measured by the time that the bacteria took to adapt to a new medium and started growing, while in simulations, *τ* was calculated from the minimum time and biomass needed for each bacterium to reproduce. There was no adaption in simulations, and the bacteria started growing right away. It is difficult to compare A due to the differences in measurements. In simulations, the measurement A is the number of bacteria, while in experiments, it is the fluorescence intensity; we elaborate more on this discrepancy in the Discussion section. Nonetheless, in general the model captures the experimental behavior of overall population growth reasonably well.

A comprehensive analysis of model accuracy would require thorough exploration of the seven-dimensional input phase space and is prohibitively expensive. Accordingly, we determine the relationship between input parameters and the model outputs by using random forest regression.

**Figure 2:**
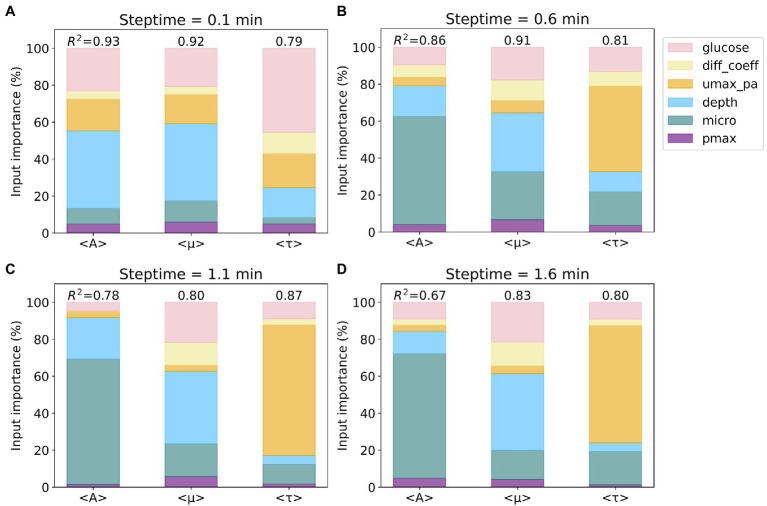
Importance of input parameters to the prediction of average values of A, *μ*, and *τ*. Four subsets based on four different steptime values were considered: steptime **(A)** 0.1, **(B)** 0.6, **(C)** 1.1, and **(D)** 1.6min. The colors of the six input parameters are shown in the legend. The *R*^2^ scores of the random forest regression models for each subset are included. Note that the importance of the six input parameters sums up to 100%.

The *R*^2^ scores and the Gini importance of the six input parameters for predicting average values of A (<A>), *μ* (<*μ*>), and *τ* (<*τ*>) for the four steptime test sets are presented in stacked bar charts (see Figure 2). We also calculated the permutation importance, and the results are similar to the Gini importance results reported herein. For the steptime of 0.1min, the *R*^2^ scores are 0.93 (<A>), 0.92 (<*μ*>), and 0.79 (<*τ*>). The three most important input parameters for predicting all three outputs are *depth*, *glucose*, and growth rate (*umax_pa*), which account for 82% of the total for <A>, 78% for <*μ*>, and 80% for <*τ*>. The importance ranking of these three inputs are the same for <A> and <*μ*>, in which *depth* is first, followed by *glucose* and *umax_pa*. Comparably, for <*τ*> *glucose* is first, followed by *umax_pa* and *depth* (Figure 2A). As the steptime increases, the *R^2^* scores for prediction of <A> and <*μ*> decrease and the score for <*τ*> increases (Figures 2B–D). This trend is most evident when comparing the *R*^2^ scores from steptime of 1.1min to those from steptime of 0.1min. For steptime of 1.1min, the *R*^2^ scores drop to 0.78 and 0.80 for <A> and <*μ*>, respectively, while the score increases to 0.87 for <*τ*> (Figure 2C). Interestingly, as the steptime increases, *microorganism* (micro) becomes one of the most important input parameters for predicting the three outputs. For example, for the steptime of 1.1min, the three most important input parameters for predicting <A> are micro, *depth*, and *glucose*, which account for 95% of the total. For <*μ*>, *depth*, *glucose*, and micro contribute to 79%, and for <*τ*>, *umax_pa*, micro, and *glucose* account for 90%.

**Figure 3:**
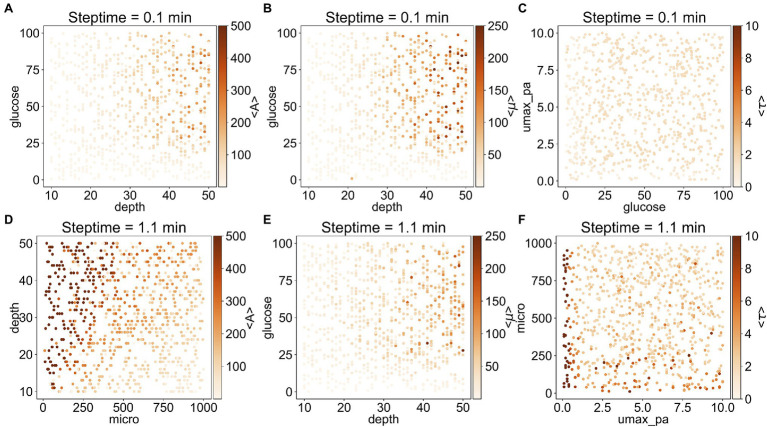
Correlation between the three outputs and their two most important inputs. **(A–C)** are the results from the subset with steptime of 0.1min and **(D–F)** are from that with steptime of 1.1min. The most important input is shown in the *x*-axis, whereas the second most important input is shown in the *y*-axis. The average values <A> **(A,D)**, <*μ*> **(B,E)**, and <*τ*> **(C,F)** are illustrated as heat maps.

To understand better the relationship between the outputs and the corresponding important input parameters, we plotted on a heat map each output as a function of the two most important inputs (Figure 3). In general, <A> and <*τ*> depend on steptime: when steptime is low (i.e., steptime of 0.1min), <A> is mostly below 300 and <*τ*> is below 2.5. However, when steptime is higher (i.e., steptime of 1.1min), <A> can adopt values above 500 and <*τ*> above 10. With steptime of 0.1min, <A> and <*μ*> are relatively low and independent of *glucose* when *depth* is less than 30. As *depth* increases, <A> and <*μ*> increase rapidly when *glucose* is above 25. <*τ*> is low regardless of *glucose* and *umax_pa* (Figures 3A–C). In comparison, with steptime of 1.1min, <A> increases rapidly as *depth* increases when micro is low but <A> increases slowly when micro is high. <*μ*> follows a similar trend here as it does with steptime of 0.1min. Overall <*τ*> is low except when *umax_pa* is 0 (Figures 3D–F).

The above results indicate that using Random Forest Regression with only three input parameters, it should be possible to predict the values of the three outputs with a *R*^2^ score of at least 0.59 (i.e., with steptime of 1.6min, the *R*^2^ score of <A> 0.67 multiplied by 88% contributed by micro, *depth*, and *glucose*). Unfortunately, this approach requires using one Random Forest Regression model per output and per steptime. It is much more convenient to predict the three outputs with one single model for all four steptime values.

**Figure 4:**
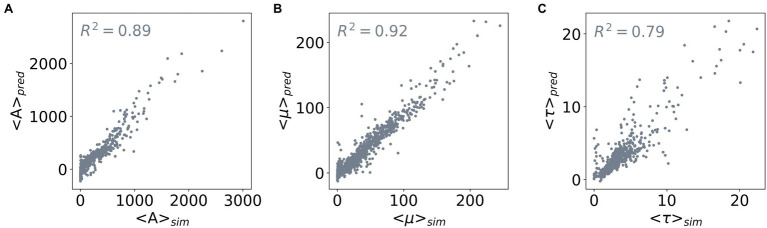
Correlation between the predicted and simulated values of the three outputs. The three outputs are **(A)** <A>, **(B)** <*μ*>, and **(C)** <*τ*>. The *x*-axis shows the average values of an output derived from the population curves (<>*_sim_*), and the *y*-axis shows the average values predicted by a feed-forward neural network (<>*_pred_*). The *R*^2^ scores of the network model used for the three output predictions are included in the subplots.

To enable predicting all three outputs with one model only, we constructed a three-layered feed-forward Neural Network. Figure 4 shows the performance of this network: it achieves *R*^2^ scores of 0.89, 0.92, and 0.79 for predicting the values of <A>, <*μ*>, and <*τ*> of the test dataset, respectively. Taken together, we demonstrated the capability of determining values of the outputs <A>, <*μ*>, and <*τ*> from the input parameters using Random Forest Regression and neural network. Based on Random Forest Regression Models, we identified important inputs for each output prediction. Besides, from a trained neural network model, we further made predictions for multiple outputs without compromising the accuracy of the model. Our results demonstrate that a metamodel derived from machine learning techniques can facilitate efficient ABM of microorganisms.

## Discussion

Traditionally, bacterial growth curves have been generated by measuring the optical density of a liquid culture over time and plotting absorbance values over time. Unfortunately, much information about how these bacterial colonies are organized and how they compete for nutrients within a given space is lost with these techniques and therefore remained not well understood. The use of ABM to model bacterial growth in a given space can yield insights into how competition for resources determines, which microbes become dominant within a certain environment. This is because ABMs of microbiology produce local and global information of microbial growth. Local information can be used to determine how different species interact, for example, by tracking the size and shape of their corresponding colonies, their boundaries, etc. Global information can inform on how a particular microbial population grows as a whole. Additionally, the application of ML to the ABM simulation results can provide insights of the important parameters of the model and further build metamodels that allow reaching length and time scales unattainable by the ABM. However, a big question is how to ensure that the ABM and the resulting metamodel of a particular microbial system is accurate. The answer is to compare the patterns that the simulated and experimental system generate at different time and length scales (Grimm et al., 2005). Extracting the patterns from a microbial system can be done by analyzing the images with a variety of ML techniques, such as those in OpenCV (Bradski, 2000). DL techniques such as segmentation (Stringer et al., 2021) and variational autoencoders (Chen et al., 2020; Kalinin et al., 2021) can then be used to reduce the dimensionality in the system and uncover trends. Applying these types of ML and DL techniques is out of the scope of this paper, but we encourage the use of such techniques in future studies to improve the accuracy of ABMs, and metamodels thereof, in microbiology. Here, we focus on gauging the accuracy of the ABM by reproducing global information of growth, e.g., the information contained in the experimental population curves. However, due to the manner in which the simulated and experimental population curves were obtained and what they measured, currently there is no appropriate method to directly compare the two results.

Experimental growth curves were generated using microscopy as a tool for measuring bacterial growth on a surface in three-dimensional space. Because this unique method does not allow us to easily quantify the number of cells present at various timepoints, we used measurement of fluorescence as a proxy for cell density. Factors that likely contribute to discrepancies between experimental curves and simulation curves include that the experimental curves were obtained by measuring the fluorescence intensity, whereas the simulated curves were generated by counting the number of bacteria directly. A direct comparison between the two requires converting the fluorescence intensity to the number of bacteria, which is likely prone to errors. Moreover, the experiments were performed in three dimensions, while only the colonies close to the surface were being imaged. In other words, bacteria growing outside the imaging plane were likely to be neglected, and thus precludes correct counting of bacteria number. Other issues, such as photobleaching of the fluorescence signal, might also affect the measurement accuracy. More effort is needed to build the bridge between simulation and experiment in microbiology, and we believe that this effort includes computer vision tools, as those in OpenCV and sophisticated DL techniques such as segmentation and VAEs. Using these techniques will allow us to obtain not only global but also local information of growth, which can then be used to gauge the accuracy of ABMs. The work we present here demonstrates a route to leverage ML and DL techniques for improved ABM development in microbiology.

## Data Availability Statement

The raw data supporting the conclusions of this article will be made available by the authors, without undue reservation.

## Author Contributions

SC and AM developed a Random Forest Regression model and a neural network to analyze the agent-based model results. AB and JM-F performed the experiments. CG, PL-L, and PA-G developed the agent-based model and the workflow for submitting the simulations. DB contributed to designing the project. MF-C designed the project and performed the simulations. All authors contributed to the article and approved the submitted version.

## Funding

DB, AB, JM-F, SC, and MF-C were supported by the Laboratory Directed Research and Development Program of Oak Ridge National Laboratory, managed by UT-Battelle, LLC, for United States Department of Energy Grant DE-AC05-00OR22725. The portion of this research regarding making the ABM model was conducted by PA-G, and PL-L at the Center for Nanophase Materials Sciences, which is a DOE Office of Science User Facility. This research used resources of the Compute and Data Environment for Science (CADES) at the Oak Ridge National Laboratory, which is supported by the Office of Science of the United States Department of Energy under Contract No. DE-AC05-00OR22725. PA-G and PL-L were supported by the Universidad Central del Ecuador (Research Project no. 26 according to RHCU.SO.08 No. 0082-2017 in official resolution with date March 21st, 2017). PL-L was also supported in part by an appointment to the Oak Ridge National Laboratory ASTRO Program, sponsored by the U.S. Department of Energy and administered by the Oak Ridge Institute for Science and Education.

## Licenses and Permissions

This manuscript has been authored by UT-Battelle, LLC under Contract No. DE-AC05-00OR22725 with the U.S. Department of Energy. The United States Government retains and the publisher, by accepting the article for publication, acknowledges that the United States Government retains a non-exclusive, paidup, irrevocable, world-wide license to publish, or reproduce the published form of this manuscript, or allow others to do so, for United States Government purposes. The Department of Energy will provide public access to these results of federally sponsored research in accordance with the DOE Public Access Plan (http://energy.gov/downloads/doe-public-access-plan).

## Conflict of Interest

The authors declare that the research was conducted in the absence of any commercial or financial relationships that could be construed as a potential conflict of interest.

## Publisher’s Note

All claims expressed in this article are solely those of the authors and do not necessarily represent those of their affiliated organizations, or those of the publisher, the editors and the reviewers. Any product that may be evaluated in this article, or claim that may be made by its manufacturer, is not guaranteed or endorsed by the publisher.

## References

[ref1] Abiodun O. I. Jantan A. Omolara A. E. Dada K. V. Mohamed N. A. Arshad H. (2018). State-of-the-art in artificial neural network applications: a survey. Heliyon 4:e00938. doi: 10.1016/j.heliyon.2018.e00938, PMID: 30519653 PMC6260436

[ref2] Araujo Granda P. Gras A. Ginovart M. (2016a). MbT-tool: an open-access tool based on thermodynamic electron equivalents model to obtain microbial-metabolic reactions to be used in biotechnological process. Comput. Struct. Biotechnol. J. 14, 325–332. doi: 10.1016/j.csbj.2016.08.001, PMID: 27635191 PMC5013251

[ref3] Araujo Granda P. Gras A. Ginovart M. Moulton V. (2016b). INDISIM-Paracoccus, an individual-based and thermodynamic model for a denitrifying bacterium. J. Theor. Biol. 403, 45–58. doi: 10.1016/j.jtbi.2016.05.017, PMID: 27179457

[ref4] Ayllón D. Railsback S. F. Vincenzi S. Groeneveld J. Almodóvar A. Grimm V. (2016). InSTREAM-Gen: modelling eco-evolutionary dynamics of trout populations under anthropogenic environmental change. Ecol. Model. 326, 36–53. doi: 10.1016/j.ecolmodel.2015.07.026

[ref5] Banitz T. Gras A. Ginovart M. (2015). Individual-based modeling of soil organic matter in NetLogo: transparent, user-friendly, and open. Environ. Model. Softw. 71, 39–45. doi: 10.1016/j.envsoft.2015.05.007

[ref6] Bergstra J. Bengio Y. (2012). Random search for hyper-parameter optimization. J. Mach. Learn. Res. 13, 281–305.

[ref7] Biau G. (2012). Analysis of a random forests model. J. Mach. Learn. Res. 13, 1063–1095.

[ref8] Bible A. N. Fletcher S. J. Pelletier D. A. Schadt C. W. Jawdy S. S. Weston D. J.. (2016). A carotenoid-deficient mutant in *Pantoea* sp. YR343, a bacteria isolated from the rhizosphere of *Populus deltoides*, is defective in root colonization. Front. Microbiol. 7:491. doi: 10.3389/fmicb.2016.00491, PMID: 27148182 PMC4834302

[ref9] Bradski G. (2000). The OpenCV library. Dr. Dobb’s J. Softw. Tools 25, 120–125.

[ref10] Chen S. H. Young M. T. Gounley J. Stanley C. Bhowmik D. (2020). Distinct structural flexibility within SARS-CoV-2 spike protein reveals potential therapeutic targets. bioRxiv: 2020.2004.2017.047548.

[ref11] Chollet F. (2015). keras, GitHub. Available at: https://github.com/fchollet/keras (Accessed September 01, 2021).

[ref12] Couronné R. Probst P. Boulesteix A.-L. (2018). Random forest versus logistic regression: a large-scale benchmark experiment. BMC Bioinformatics 19:270. doi: 10.1186/s12859-018-2264-5, PMID: 30016950 PMC6050737

[ref13] Cregger M. Carper D. L. Christel S. Doktycz M. Labbe J. Michener J.. (2021). Plant-microbe interactions: from genes to ecosystems using Populus as a model system. Phytobiomes J. 5, 29–38. doi: 10.1094/PBIOMES-01-20-0009-FI

[ref14] Font Marques M. Ginovart M. (2016). Modelización de crecimientos microbianos en medios heterogéneos y de movilidad reducida. Model. Sci. Educ. Learn. 9, 81–120. doi: 10.4995/msel.2016.5789

[ref15] Ginovart M. Cañadas J. C. (2008). INDISIM-YEAST: an individual-based simulator on a website for experimenting and investigating diverse dynamics of yeast populations in liquid media. J. Ind. Microbiol. Biotechnol. 35:1359. doi: 10.1007/s10295-008-0436-4, PMID: 18726624

[ref16] Ginovart M. López D. Valls J. (2002). INDISIM, an individual-based discrete simulation model to study bacterial cultures. J. Theor. Biol. 214, 305–319. doi: 10.1006/jtbi.2001.2466, PMID: 11812180

[ref17] Gras A. Ginovart M. Valls J. Baveye P. C. (2011). Individual-based modelling of carbon and nitrogen dynamics in soils: parameterization and sensitivity analysis of microbial components. Ecol. Model. 222, 1998–2010. doi: 10.1016/j.ecolmodel.2011.03.009

[ref18] Grimm V. Berger U. DeAngelis D. L. Polhill J. G. Giske J. Railsback S. F. (2010). The ODD protocol: a review and first update. Ecol. Model. 221, 2760–2768. doi: 10.1016/j.ecolmodel.2010.08.019

[ref19] Grimm V. Railsback S. F. Vincenot C. E. Berger U. Gallagher C. DeAngelis D. L.. (2020). The ODD protocol for describing agent-based and other simulation models: a second update to improve clarity, replication, and structural realism. J. Artif. Soc. Soc. Simul. 23:7. doi: 10.18564/jasss.425933204215

[ref20] Grimm V. Revilla E. Berger U. Jeltsch F. Mooij W. M. Railsback S. F.. (2005). Pattern-oriented modeling of agent-based complex systems: lessons from ecology. Science 310:987. doi: 10.1126/science.1116681, PMID: 16284171

[ref21] Gunaratne C. Garibay I. (2018). NL4Py: Agent-based modeling in python with parallelizable NetLogo workspaces. arXiv [preprint] arXiv:1808.03292.

[ref22] Hellweger F. L. Clegg R. J. Clark J. R. Plugge C. M. Kreft J.-U. (2016). Advancing microbial sciences by individual-based modelling. Nat. Rev. Microbiol. 14, 461–471. doi: 10.1038/nrmicro.2016.62, PMID: 27265769

[ref23] Hellweger F. L. Kravchuk E. S. Novotny V. Gladyshev M. I. (2008). Agent-based modeling of the complex life cycle of a cyanobacterium (Anabaena) in a shallow reservoir. Limnol. Oceanogr. 53, 1227–1241. doi: 10.4319/lo.2008.53.4.1227

[ref24] Kalinin S. V. Zhang S. Valleti M. Pyles H. Baker D. De Yoreo J. J.. (2021). Disentangling rotational dynamics and ordering transitions in a system of self-organizing protein nanorods via rotationally invariant latent representations. ACS Nano 15, 6471–6480. doi: 10.1021/acsnano.0c08914, PMID: 33861068

[ref25] Kapetas L. Ngwenya B. T. MacDonald A. M. Elphick S. C. (2012). Thermodynamic and kinetic controls on cotransport of pantoea agglomerans cells and Zn through clean and iron oxide coated sand columns. Environ. Sci. Technol. 46, 13193–13201. doi: 10.1021/es302801a, PMID: 23153272

[ref26] Kreft J.-U. Booth G. Wimpenny J. W. T. (1998). BacSim, a simulator for individual-based modelling of bacterial colony growth. Microbiology 144, 3275–3287. doi: 10.1099/00221287-144-12-3275, PMID: 9884219

[ref27] Kreft J.-U. Plugge C. M. Prats C. Leveau J. H. J. Zhang W. Hellweger F. L. (2017). From genes to ecosystems in microbiology: modeling approaches and the importance of individuality. Front. Microbiol. 8:2299. doi: 10.3389/fmicb.2017.02299, PMID: 29230200 PMC5711835

[ref28] Lee J.-Y. Sadler N. C. Egbert R. G. Anderton C. R. Hofmockel K. S. Jansson J. K.. (2020). Deep learning predicts microbial interactions from self-organized spatiotemporal patterns. Comput. Struct. Biotechnol. J. 18, 1259–1269. doi: 10.1016/j.csbj.2020.05.023, PMID: 32612750 PMC7298420

[ref29] Leveau J. H. J. Hellweger F. L. Kreft J.-U. Prats C. Zhang W. (2018). Editorial: the individual microbe: single-cell analysis and agent-based modelling. Front. Microbiol. 9:2825. doi: 10.3389/fmicb.2018.02825, PMID: 30519230 PMC6258959

[ref30] Li B. Taniguchi D. Gedara J. P. Gogulancea V. Gonzalez-Cabaleiro R. Chen J.. (2019). NUFEB: a massively parallel simulator for individual-based modelling of microbial communities. PLoS Comput. Biol. 15:e1007125. doi: 10.1371/journal.pcbi.1007125, PMID: 31830032 PMC6932830

[ref31] Ligmann-Zielinska A. (2013). Spatially-explicit sensitivity analysis of an agent-based model of land use change. Int. J. Geogr. Inf. Sci. 27, 1764–1781. doi: 10.1080/13658816.2013.782613

[ref32] Ligmann-Zielinska A. Kramer D. B. Spence Cheruvelil K. Soranno P. A. (2014). Using uncertainty and sensitivity analyses in socioecological agent-based models to improve their analytical performance and policy relevance. PLoS One 9:e109779. doi: 10.1371/journal.pone.0109779, PMID: 25340764 PMC4207681

[ref33] McCarty P. L. (2007). Thermodynamic electron equivalents model for bacterial yield prediction: modifications and comparative evaluations. Biotechnol. Bioeng. 97, 377–388. doi: 10.1002/bit.21250, PMID: 17089390

[ref34] Pedregosa F. Varoquaux G. Gramfort A. Michel V. Thirion B. Grisel O.. (2011). Scikit-learn: machine learning in python. J. Mach. Learn. Res. 12, 2825–2830. doi: 10.5555/1953048.2078195

[ref35] Pereda M. Santos J. I. Galán J. M. (2017). “A brief introduction to the use of machine learning techniques in the analysis of agent-based models,” in Advances in Management Engineering. ed. Hernández C. (Cham: Springer International Publishing), 179–186.

[ref36] Portell X. Gras A. Ginovart M. (2014). INDISIM-Saccha, an individual-based model to tackle *Saccharomyces cerevisiae* fermentations. Ecol. Model. 279, 12–23. doi: 10.1016/j.ecolmodel.2014.02.007

[ref37] Prats C. Ferrer J. Gras A. Ginovart M. J. M. (2010). Individual-based modelling and simulation of microbial processes: yeast fermentation and multi-species composting. Math. Comput. Model. Dyn. Syst. 16, 489–510. doi: 10.1080/13873954.2010.481809

[ref38] Railsback S. F. Ayllón D. Berger U. Grimm V. Lytinen S. Sheppard C.. (2017). Improving execution speed of models implemented in NetLogo. J. Artif. Soc. Soc. Simul. 20:3. doi: 10.18564/jasss.3282

[ref39] Stringer C. Wang T. Michaelos M. Pachitariu M. (2021). Cellpose: a generalist algorithm for cellular segmentation. Nat. Methods 18, 100–106. doi: 10.1038/s41592-020-01018-x, PMID: 33318659

[ref40] ten Broeke G. van Voorn G. Ligtenberg A. (2016). Which sensitivity analysis method should I use for my agent-based model? J. Artif. Soc. Soc. Simul. 19:5 doi: 10.18564/jasss.2857

[ref41] Thiele J. C. Kurth W. Grimm V. (2014). Facilitating parameter estimation and sensitivity analysis of agent-based models: a cookbook using NetLogo and ‘R’. J. Artif. Soc. Soc. Simul. 17:11. doi: 10.18564/jasss.2503

[ref42] Virtanen P. Gommers R. Oliphant T. E. Haberland M. Reddy T. Cournapeau D.. (2020). SciPy 1.0: fundamental algorithms for scientific computing in python. Nat. Methods 17, 261–272. doi: 10.1038/s41592-019-0686-2, PMID: 32015543 PMC7056644

[ref43] Wilensky U. (1999). NetLogo. Evanston, IL: Centre for Connected Learning and Computer-Based Modeling, Northwestern University. Available at: http://ccl.northwestern.edu/netlogo/

[ref44] Wilmoth J. L. Doak P. W. Timm A. Halsted M. Anderson J. D. Ginovart M.. (2018). A microfluidics and agent-based modeling framework for investigating spatial organization in bacterial colonies: the case of *Pseudomonas aeruginosa* and H1-type VI secretion interactions. Front. Microbiol. 9:33. doi: 10.3389/fmicb.2018.00033, PMID: 29467721 PMC5808251

[ref45] Wushensky J. A. Youngster T. Mendonca C. M. Aristilde L. (2018). Flux connections between gluconate pathway, glycolysis, and pentose-phosphate pathway during carbohydrate metabolism in *Bacillus megaterium* QM B1551. Front. Microbiol. 9:2789. doi: 10.3389/fmicb.2018.02789, PMID: 30524402 PMC6262346

[ref46] Zwietering M. H. Jongenburger I. Rombouts F. M. van’t Riet K. (1990). Modeling of the bacterial growth curve. Appl. Environ. Microbiol. 56:1875. doi: 10.1128/aem.56.6.1875-1881.1990, PMID: 16348228 PMC184525

